# Effects of high-intensity treadmill exercise on GLUT4, TNF-α, and INSIG1 mRNA expression and blood glucose levels in streptozotocin-induced diabetic rats

**DOI:** 10.3389/fendo.2026.1973677

**Published:** 2026-10-08

**Authors:** Sezgin Hepsert, Semih Dalkılıç, Lütfiye Kadıoğlu Dalkılıç, İbrahim Aydınlı, Dilek Arslan, Mehmet Akif Sarı, Yakup Kılıc

**Affiliations:** 1 Physical Education Teaching, Ministry of Education, Elazig, Türkiye; 2 Department of Biology, Faculty of Science, Firat University, Elazig, Türkiye; 3 Faculty of Sports Sciences, Kocaeli University, Kocaeli, Türkiye; 4 Baskil Vacational School, Firat University, Elazig, Türkiye; 5 Department of Physical Education and Sports, Fırat University, Elazig, Türkiye

**Keywords:** exercise, gene expression, GLUT4, INSIG1, STZ-induced diabetes, TNF-α

## Abstract

**Background:**

Exercise plays a central role in diabetes management by modulating glucose homeostasis and metabolic gene networks. This study investigated the effects of a 6-week high-intensity treadmill exercise protocol on GLUT4, TNF-α, and INSIG1 mRNA expression in soleus muscle and blood glucose levels in streptozotocin (STZ)-induced diabetic rats.

**Methods:**

In this randomized experimental study, 45 eight-week-old male Sprague-Dawley rats were allocated to Control (C), Sedentary diabetic (S), and Exercise diabetic (E) groups (n=15/group). Diabetes was induced via a single intraperitoneal STZ injection (60 mg/kg) and confirmed 72 h post-injection (blood glucose >200 mg/dL). Group E underwent treadmill exercise (30 min/day, 5 days/week, 6 weeks). Blood glucose was assessed longitudinally, and soleus muscle GLUT4, TNF-a, and INSIG1 mRNA expression levels were analyzed post-intervention via qRT-PCR.

**Results:**

Blood glucose levels were 80.33 ± 7.79, 86.33 ± 7.94, and 88.27 ± 6.52 mg/dL at weeks 1, 3, and 6, respectively, in the C group; 356.64 ± 160.63, 361.91 ± 133.09, and 362.64 ± 116.10 mg/dL in the S group; and 452.13 ± 165.34, 376.73 ± 189.70, and 286.80 ± 145.92 mg/dL in the E group. Mixed-effects analysis revealed significant main effects of group (F(2,38) = 29.520, p < 0.001, ηp² = 0.608) and time (F(2,76) = 6.651, p = 0.022, ηp² = 0.149), and a significant group × time interaction (F(4,76) = 9.143, p < 0.001, ηp² = 0.325). Although blood glucose decreased progressively in E, post-test levels did not differ significantly between E and S. GLUT4 mRNA expression was significantly higher, whereas TNF-α mRNA expression was significantly lower in E than S (p < 0.05). No significant difference was observed in INSIG1 mRNA expression between E and S (p > 0.05).

**Conclusion:**

A 6-week high-intensity treadmill exercise regimen was associated with significant GLUT4 upregulation and reduced TNF-α mRNA expression in soleus muscle, without significant post-intervention blood glucose differences compared with sedentary diabetic controls. These findings suggest localized transcriptional adaptations rather than restoration of systemic glucose homeostasis or superior glycemic control under STZ-induced diabetic conditions.

## Introduction

Diabetes mellitus is a metabolic condition characterized by elevated blood glucose levels resulting from impaired insulin secretion or action (1, 2). Ranked among the leading causes of death globally, diabetes severely reduces quality of life while imposing significant economic burdens on individuals, families, and healthcare systems. According to recent global estimates, approximately 537 million adults were living with diabetes in 2021, a figure projected to rise to 783 million by 2045 (3). Consequently, diabetes is now recognized as one of the most critical public health challenges of the 21st century, prompting extensive research into both preventive and therapeutic strategies (4–6). Standard management relies on anti-diabetic medications, pharmacological agents, recombinant insulin, and regular exercise interventions (7–9). Among these, exercise serves as a cornerstone therapy by enhancing glycemic control, blood lipid profiles, and endothelial function (10). Beyond its systemic metabolic benefits, exercise induces critical adaptations in skeletal muscle, the primary site of glucose disposal and a central determinant of glucose homeostasis.

There are studies in the literature examining the course of experimental diabetes through the response of different markers to exercise (11). Among these, GLUT4 enables glucose transport into cells and is densely expressed in skeletal muscle tissue. Muscle contraction during exercise stimulates glucose uptake through signaling pathways that are, at least partly, independent of insulin signaling (12). This mechanism is particularly relevant in STZ-induced diabetes, where insulin-mediated glucose transport is impaired, allowing exercise to partially overcome defects in insulin action. Furthermore, chronic exercise training increases skeletal muscle GLUT4 mRNA expression and improves overall glucose disposal, highlighting GLUT4 as a crucial molecular target for understanding exercise-induced metabolic adaptations in diabetes.

TNF-α, which is described as an adipokine, is closely related to glucose regulation, lipid metabolism, and cardiovascular function (13). Additionally, it is an effective pro-inflammatory cytokine associated with insulin resistance and metabolic dysfunction in experimental models of diabetes (14). TNF-α levels are generally high in obesity, diabetes, and cardiovascular diseases. It has been stated that regular exercise has a down-regulation effect on the TNF-α level in muscle tissue. It is also known that the type, intensity, and duration of exercise are determinants of the target response (15). It has been suggested that this effect is due to myokines such as IL-6 secreted from active muscles during exercise, reducing the inflammatory effects of TNF-α and creating a systemic anti-inflammatory effect (16). However, the increased TNF-α mRNA expression observed in the Exercise group in the present study should be interpreted cautiously. An increase in TNF-α mRNA expression in skeletal muscle does not necessarily indicate a detrimental inflammatory adaptation, worsening insulin resistance, or a systemic pro-inflammatory response. The biological interpretation of TNF-α may depend on the tissue examined, timing of measurement, exercise intensity, cellular source, and the level at which the response is assessed, such as mRNA, protein, or circulating levels. Therefore, the increased TNF-α mRNA expression observed in the soleus muscle may represent a tissue-specific or exercise-induced transcriptional response rather than evidence of a systemic inflammatory response.

INSIG1 mRNA expression plays a critical role in regulating lipid and glucose metabolism, with alterations in its expression pattern often accompanying metabolic imbalances (17). Given that STZ-induced diabetes impairs insulin signaling and metabolic homeostasis, insulin-regulated genes such as INSIG1 are involved in complex mechanisms underlying blood glucose and lipid control (18). Because skeletal muscle represents a major metabolically active site for glucose utilization, shifts in key homeostatic genes like INSIG1 may reflect or modulate tissue-specific metabolic adaptations to physical activity. Increased energy demand during exercise may influence INSIG1 activity, which could be linked to altered lipid and glucose dynamics under diabetic conditions. However, given the context-dependent physiological functions of INSIG1, further research is required to clarify how its regulation in skeletal muscle interacts with exercise-induced metabolic adaptations in diabetes (19).

Although GLUT4, TNF-α, and INSIG1 have individually been associated with glucose transport, inflammation, and metabolic regulation, respectively, their coordinated response to exercise in skeletal muscle under diabetic conditions remains insufficiently characterized. Skeletal muscle metabolic adaptations under diabetic conditions operate through an interconnected network where TNF-α-mediated inflammatory signaling and INSIG1-regulated lipid homeostasis directly interact to influence the cellular environment governing GLUT4 expression and muscle glucose transport. In particular, investigating these interlinked molecular targets within the same experimental model provides a more integrated biological perspective on whether exercise-related improvements in glycemic control are accompanied by coordinated alterations across skeletal muscle glucose transport, inflammatory signaling, and lipid metabolic regulation.

In this context, the current study aims to examine the relationship between regular exercise and GLUT4, TNF-α, and INSIG1 mRNA expression in the soleus muscle tissue of STZ-induced diabetic rats. In this study, these target markers, which have been investigated separately in relation to metabolic regulation and diabetes, were examined concurrently using a standardized exercise protocol with fixed duration, frequency, and intensity to investigate exercise-related molecular responses under STZ-induced diabetic conditions.

## Materials and methods

### Experimental animals and housing conditions

The experimental animals used in this study were 8-week-old male Sprague-Dawley rats obtained from the Fırat University Experimental Animal Production and Research Center (FÜDAM). The rats were housed in wire-topped polypropylene cages under controlled environmental conditions, with a room temperature of 22 ± 2 °C, relative humidity of 35–40%, a noise level below 85 dB, and a light intensity of 40 lux, under a 12-hour light/12-hour dark photoperiod. All animals had ad libitum access to standard laboratory chow and water throughout the experimental period. The rats were selected to be of similar age, genetic background, and health status to minimize potential biological variability among experimental groups. The experimental procedures were conducted in accordance with the guidelines of the ARRIVE guidelines and were approved by the Fırat University Animal Experiments and Local Ethics Committee (Approval No. 2020/10). The study was reported in accordance with the ARRIVE guidelines.

### Design of the study

An *a priori* sample size calculation was performed using G*Power 3.1.9.7 (Franz Faul, Universität Kiel, Germany). The sample size estimation was based on a one-way analysis of variance (ANOVA) for comparison of the three experimental groups, with an assumed effect size of 0.45, a type I error rate (α) of 0.05, and a statistical power (1−β) of 0.90. The primary outcome considered for the sample size calculation was GLUT4 mRNA expression, which was considered the principal molecular endpoint reflecting exercise-related adaptations in skeletal muscle glucose metabolism. The effect size (f = 0.45) was specified *a priori* for the power analysis. Based on these assumptions, the minimum required sample size was determined as 15 rats per group, resulting in a total of 45 rats. Although multiple molecular outcomes were evaluated in the study, GLUT4 mRNA expression was considered the primary molecular endpoint for the purpose of sample size estimation. The animals were randomly allocated to three groups: Control (C), Sedentary (S), and Exercise (E), with 15 rats in each group. All 45 randomized animals completed the experimental protocol, and no animals died, were excluded, or were lost to follow-up during the study. Therefore, all 45 animals contributed to the final analyses. The investigators responsible for tissue processing and qRT-PCR analyses were blinded to group allocation during the laboratory analyses. Diabetes was induced in the S and E groups by a single intraperitoneal injection of streptozotocin (STZ) at a dose of 60 mg/kg, dissolved in 0.1 M citrate buffer (pH 4.5) (20). A tail-vein blood glucose concentration >200 mg/dL was considered indicative of diabetes (21), and diabetes was confirmed 72 h after STZ administration. Blood glucose levels were measured at the 1st, 3rd, and 6th weeks of the experimental period following a 6-hour fasting period, with free access to water, to ensure standardized metabolic conditions. The E group underwent high-intensity treadmill exercise (MAY-TME 0804, Commat Limited) between 08:00 and 10:00 for 6 consecutive weeks, 5 days per week. The rats performed continuous treadmill running for 30 min per session at a speed of 1.8 km/h (30 m/min) with a fixed inclination of 15°. The same exercise intensity and duration were maintained throughout the entire six-week intervention period, with no separate adaptation period or week-by-week progression. At the end of the sixth week, the rats were deeply anesthetized with ketamine (90 mg/kg) and xylazine (10 mg/kg), administered intraperitoneally. After loss of reflexes and complete loss of consciousness, the rats were euthanized by cervical dislocation (22).

According to **Table 1**, it is seen that the rats were assigned to the groups randomly and in equal numbers.

**Table 1:** Random assignment of rats to groups.

Variable	Rat–Group Assignments									
Subject	1	2	3	4	5	6	7	8	9	10
Groups	E	S	E	S	E	S	S	C	S	E
Subject	11	12	13	14	15	16	17	18	19	20
Groups	E	C	E	S	C	S	C	C	E	C
Subject	21	22	23	24	25	26	27	28	29	30
Groups	C	S	C	C	S	C	E	C	S	E
Subject	31	32	33	34	35	36	37	38	39	40
Groups	S	C	S	E	S	E	S	S	E	E
Subject	41	42	43	44	45					
Groups	C	C	E	E	C					

Note: Subject ID indicates the identification number assigned to each rat, and group assignment indicates the group to which each rat was randomly allocated. E, exercise group; S, sedentary group; C, control group.

### Inclusion and exclusion criteria

Male Wistar albino rats aged 8 weeks and weighing 200–250 g with healthy physiological characteristics were selected as the inclusion criterion for the study. This selection aims to minimize genetic and age-related variables by ensuring that the experiment is conducted on a homogeneous animal model. Exclusion criteria included the development of disease during the experiment, excessive weight loss (>15–20%), restricted movement, behavioral abnormalities, post-injection complications, and failure to comply with the specified exercise protocols. These criteria were established to keep experimental variables under control and increase the reliability of the results. Furthermore, to protect animal welfare and comply with ethical guidelines, all observations were regularly recorded, and any animal exhibiting adverse health conditions was removed from the study (23).

### Exercise training

The treadmill exercise (MAY-TME 0804, Commat Limited) was performed for six consecutive weeks, five days per week, between 08:00 and 10:00 each day. The high-intensity treadmill exercise protocol was applied only to the Exercise (E) group. Rats in this group performed continuous treadmill running for 30 min per session at a speed of 1.8 km/h (equivalent to 30 m/min) and a fixed inclination of 15°. The same exercise intensity and duration were maintained throughout the six-week intervention period, with no separate adaptation period or additional week-by-week progression. This exercise intensity was selected based on previously published rodent treadmill protocols reporting associations between treadmill speed, inclination, and relative exercise intensity. Previous studies have reported that treadmill exercise performed at lower speeds and without inclination may correspond to lower relative exercise intensities, whereas higher treadmill speeds combined with an incline have been associated with substantially greater relative exercise intensity (24, 25). Because oxygen consumption, blood lactate, respiratory exchange ratio, and other direct physiological indicators of exercise intensity or anaerobic metabolism were not measured in the present study, the intervention was classified as high-intensity treadmill exercise rather than being definitively characterized as anaerobic.

### Collection of tissues

Rats were euthanized using an ethical protocol, and soleus muscle tissues were obtained. To ensure RNA stability, these tissues were immediately transferred to RNA Later^®^ solution. Specifically, RNA Later^®^ solution rapidly penetrates tissues, inactivating RNA and preserving RNA integrity during storage. Tissue samples were incubated at 4 °C for one day to ensure adequate penetration of the solution into the tissues. After incubation, excess RNA Later^®^ solution was discarded, and the samples were stored at −20 °C. Within two weeks, total RNA isolation was performed from the tissue samples. TRIzol^®^ or similar reagents are commonly used for RNA isolation. This method ensures high purity and high yield of RNA and is widely used to improve the reliability and efficiency of RNA isolation (26).

### RNA isolation from tissue samples

RNA isolation from rat soleus muscle tissue was performed using the Invitrogen PureLink™ RNA Mini Kit. Approximately 100 mg of tissue sample was crushed on ice and transferred to 2 mL Eppendorf tubes, with 1000 µL of TRIzol^®^ and 5 µL of DTT added to each tube. A measure of zirconium beads was added to the samples, which were vortexed for 5 minutes to homogenize. The samples were then vortexed at 5-minute intervals and incubated on ice for a total of 15 minutes. Following incubation, the samples were centrifuged at 14,000 rpm at 4 °C for 10 minutes, and the supernatants were transferred to separate Eppendorf tubes, each containing 200 µL of chloroform. The tubes were vortexed until a color change was observed and incubated on ice for an additional 10 minutes, resulting in three phases: DNA in the lower phase, protein in the middle phase, and RNA in the upper phase. The upper phase containing RNA was transferred to another tube and diluted with isopropyl alcohol at a 1:1 volume ratio, vortexed for 3–5 minutes, and incubated on ice for 10 minutes. The samples were centrifuged at 14,000 rpm at 4 °C for 15 minutes, the supernatant was completely removed, and 1 mL of 75% ethanol was added to the pellet and vortexed. The pellet was then centrifuged for an additional 5 minutes at 14,000 rpm, the pellet was removed, and the tubes were incubated on ice for 6 minutes with the caps open. Finally, diethylpyrocarbonate (DEPC) was added based on the RNA amount, and the RNA solution was kept on ice for 15–20 minutes (27).

### Quality and control of RNAs

The isolated RNA samples were subjected to electrophoresis for 20–30 minutes on 1% agarose gels containing 5 µL RedSafe dye. RNA integrity was qualitatively assessed using an Invitrogen iBright 750 UV gel imaging system by examining the presence and appearance of 18S and 28S rRNA bands. RNA samples considered suitable based on this qualitative assessment were selected for cDNA synthesis. In addition, RNA concentrations were measured using the Qubit HS RNA Test Kit on a Qubit fluorometer, and the measured RNA concentration was used as the starting concentration for cDNA synthesis. cDNA synthesis was performed using Applied Biosystems High Capacity cDNA Reverse Transcription Kits with a 10-minute pre-incubation at 25 °C, 120 minutes of reverse transcription at 37 °C, enzyme inactivation at 85 °C for 5 minutes, and cooling at 4 °C for 4 minutes (28).

### Quantitative real-time (qRT)-PCR

Following cDNA synthesis, gene expression levels of GLUT4 (Slc2a4), TNF-α (Tnf), and INSIG1 were determined using quantitative real-time PCR (qRT-PCR). GAPDH (glyceraldehyde-3-phosphate dehydrogenase) was used as the reference gene for normalization of target gene expression. Reactions were performed using an Applied Biosystems StepOnePlus Real-Time PCR System (Applied Biosystems, USA). The amplification protocol consisted of an initial denaturation step at 95 °C for 3 min, followed by amplification cycles of 95 °C for 15 s, 54 °C for 10 s, and 72 °C for 15 s. Following amplification, a dissociation/melting analysis was performed at 95 °C for 20 s, 60 °C for 1 min, and 95 °C for 15 s to assess amplification specificity. The primer sequences and expected amplicon sizes for the target and reference genes are presented in **Table 2**.

**Table 2:** Primer sequences and expected amplicon sizes used for qRT-PCR.

Gene	Gene symbol	Forward primer (5′–3′)	Reverse primer (5′–3′)	Expected amplicon (bp)
GAPDH	*Gapdh*	GCAAGTTCAACGGCACAGTCAAG	CTACTCAGCACCAGCATCACCC	118
GLUT4	*Slc2a4*	GCTTCTTCCTGGAAGCTCTCTAC	AAGAGCTGATCCTGTCCCCAA	142
TNF-α	*Tnf*	GCTCCTCTCTCAAGTCAGCAA	GTCCCAGCATCTTGTGACCT	155
INSIG1	*Insig1*	ACTTGGTGGCCACTATCCTG	GGTAAGCAAACGACCGAGAT	126

Primer sequences are presented in the 5′–3′ direction. Amplicon sizes represent the expected product lengths.

All qRT-PCR reactions were performed in triplicate technical replicates for each biological sample, and the mean Ct value of the technical replicates was used for subsequent expression analyses. No-template controls (NTC), in which cDNA was replaced with nuclease-free water, were included to monitor potential contamination and nonspecific amplification. No-reverse-transcription (no-RT) controls were also included to assess potential genomic DNA contamination during the cDNA synthesis process. Relative mRNA expression levels were normalized to GAPDH expression and expressed as fold change relative to the Control group. The expression levels were analyzed using the Relative mRNA Expression algorithm, and changes were expressed as fold change (Fc) (28). The same qRT-PCR conditions were applied to all experimental samples. Detailed information regarding the reaction composition and experimentally determined amplification efficiency could not be verified from the original experimental records. Therefore, these parameters were not retrospectively inferred from generic qRT-PCR protocols or secondary sources.

### Statistical analysis

The data obtained were analyzed using SPSS version 22.0 (IBM, Armonk, NY, USA), and GraphPad Prism 8 was used for data visualization. The normality of the data distribution was assessed based on skewness and kurtosis values, with values between −2 and +2 considered indicative of an approximately normal distribution (29). Blood glucose levels were assessed at three time points (pre-test, mid-test, and post-test) and analyzed using a mixed-effects model (REML), with time as the within-subject factor and experimental group as the between-subject factor. The group × time interaction was examined to determine whether changes in blood glucose levels over time differed among the experimental groups. Partial eta squared (ηp²) was reported as the effect size for the fixed effects of the mixed-effects model. For comparisons of post-test molecular outcomes among the three experimental groups, one-way ANOVA was performed. When a significant overall group effect was detected, Bonferroni-adjusted *post-hoc* pairwise comparisons were performed to determine which groups differed from each other. Effect sizes for between-group comparisons of molecular outcomes were calculated according to Cohen’s d. An effect size of 0.2 was considered small, 0.5 medium, and 0.8 large (30). The significance level was set at p < 0.05 for all statistical tests.

## Results

Blood glucose levels were evaluated at weeks 1, 3, and 6 in the Control (C), Sedentary diabetic (S), and Exercise diabetic (E) groups. The mixed-effects model (REML) revealed a significant main effect of group (F(2, 38) = 29.520, p < 0.001, ηp² = 0.608) and a significant main effect of time (F(2, 76) = 6.651, p = 0.022, ηp² = 0.149). Importantly, a significant group × time interaction was observed (F(4, 76) = 9.143, p < 0.001, ηp² = 0.325), indicating that the pattern of change in blood glucose levels over time differed among the experimental groups. As shown in **Table 3**, blood glucose levels remained relatively stable in the Control and Sedentary diabetic groups, whereas the Exercise group showed a progressive decrease from week 1 to week 6.

**Table 3:** Blood glucose levels and mixed-effects model results.

Group	Week 1	Week 3		Week 6
Control	80.33 ± 7.79	86.33 ± 7.94		88.27 ± 6.52
Sedentary	356.64 ± 160.63	361.91 ± 133.09		362.64 ± 116.10
Exercise	452.13 ± 165.34	376.73 ± 189.70		286.80 ± 145.92
Effect	F	df	p	Partial η²
Group	29.520	2, 38	0.001	0.608
Time	6.651	2, 76	0.022	0.149
Group × Time	9.143	4, 76	0.001	0.325
Group	29.520	2, 38	0.001	0.608

**Figure 1:**
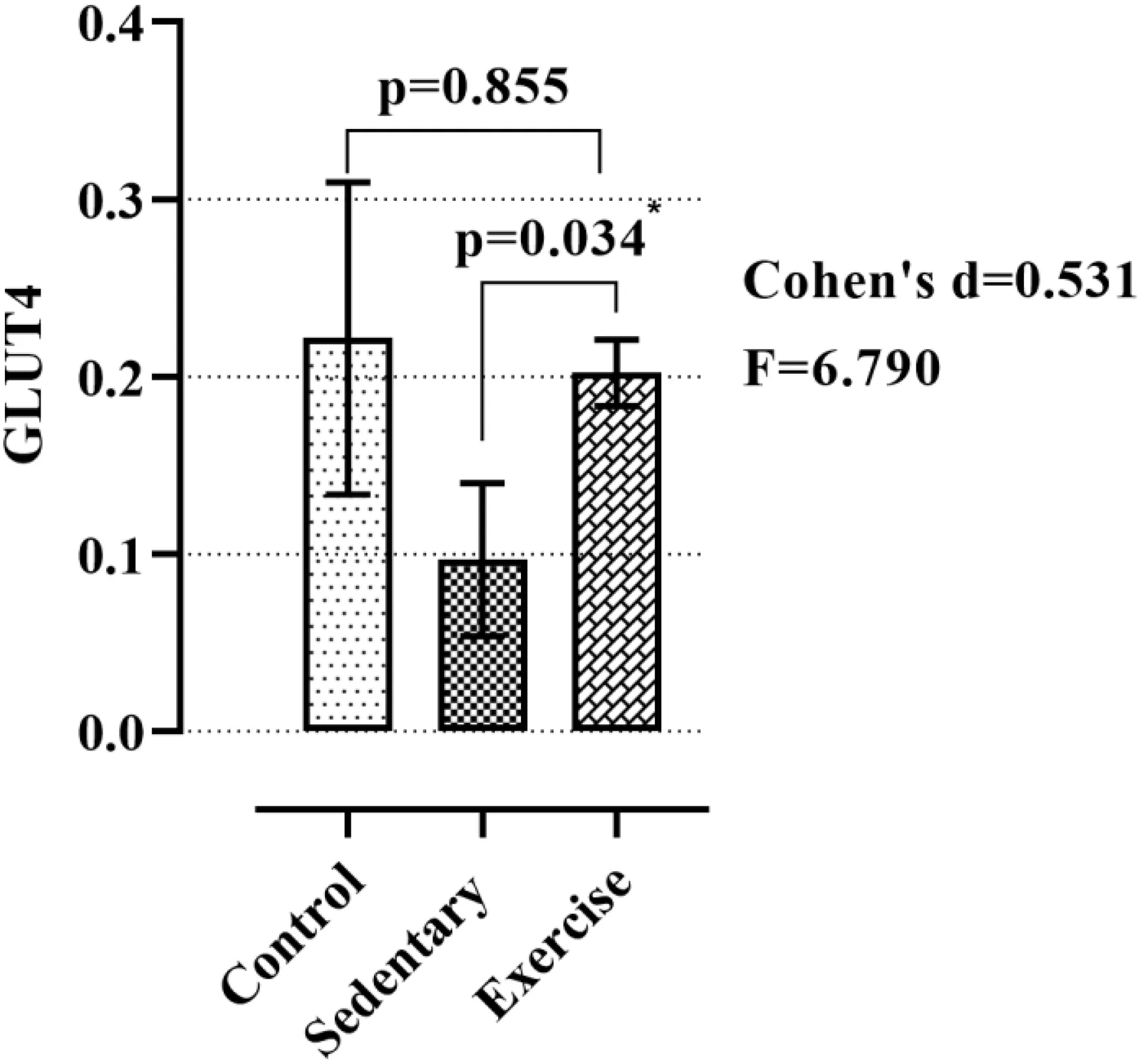
GLUT4 mRNA expression analysis across experimental groups C, Control; S, Sedentary; E, Exercise. Individual data points are displayed alongside group summary values presented as Mean ± SD (Control: 0.222 ± 0.088, Sedentary: 0.097 ± 0.043, Exercise: 0.202 ± 0.019; n = 15 rats per group). Statistical significance was evaluated using one-way ANOVA followed by Bonferroni’s *post-hoc* test for multiple comparisons. *p < 0.05 indicates a statistically significant upregulation in the Exercise group compared with the Sedentary group (p < 0.05, mean difference: -0.1054, 95% CI: -0.2068 to -0.0040), whereas no significant difference was observed between the Control and Exercise groups (p > 0.05) (**Figure 1**).

**Figure 2:**
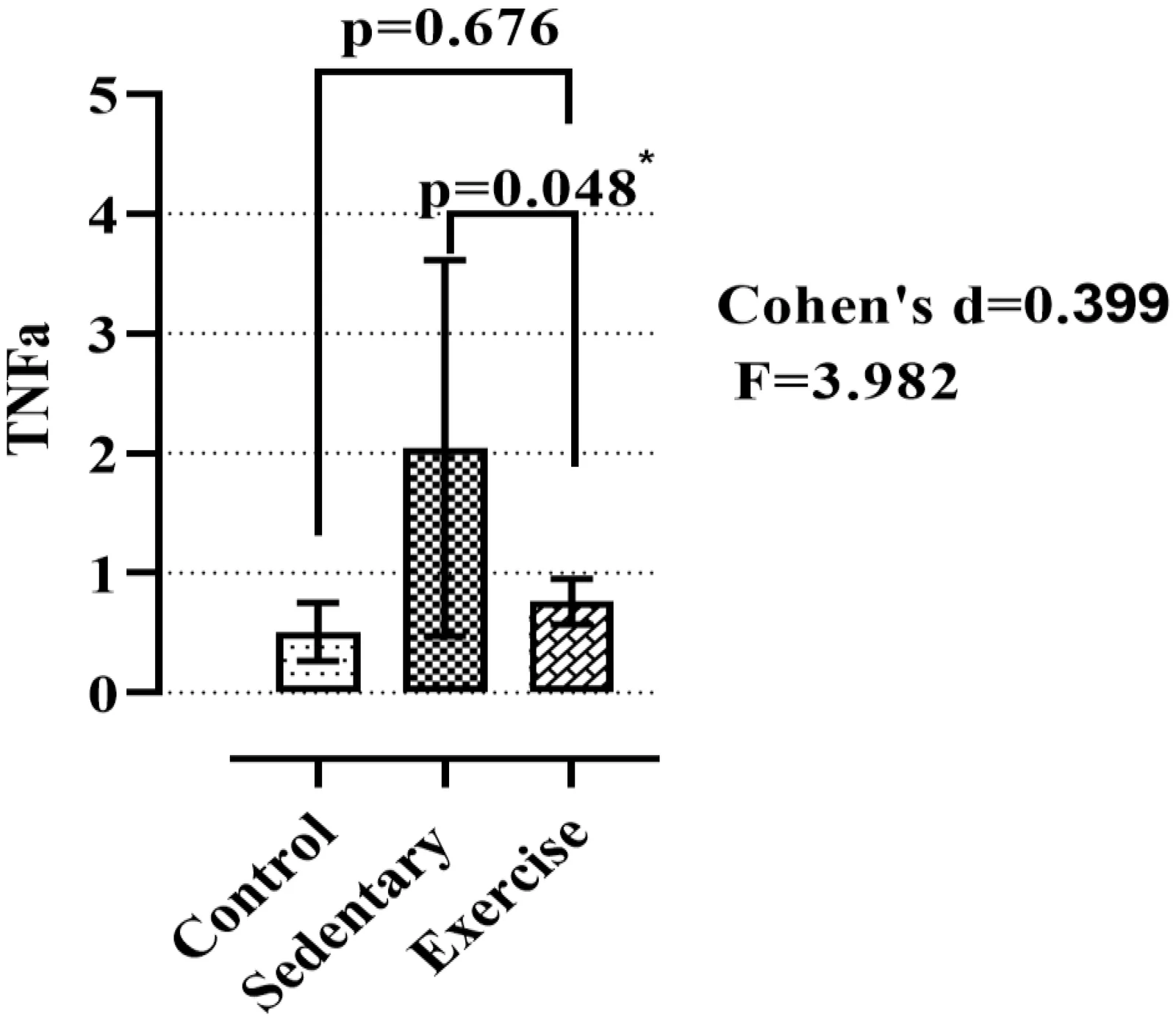
TNF-α mRNA expression analysis across experimental groups C, Control; S, Sedentary; E, Exercise. Individual data points are displayed alongside group summary values presented as Mean ± SD (Control: 0.509 ± 0.245, Sedentary: 2.044 ± 1.567, Exercise: 0.760 ± 0.190; n = 15 rats per group). Statistical significance was evaluated using one-way ANOVA followed by Bonferroni’s *post-hoc* test for multiple comparisons. *p < 0.05 indicates a statistically significant downregulation of TNF-α mRNA expression in the Exercise group compared with the Sedentary group (p < 0.05, mean difference [S − E]: 1.284, 95% CI: 0.3375 to 2.906), whereas no significant difference was observed between the Control and Exercise groups (p > 0.05) (**Figure 2**).

**Figure 3:**
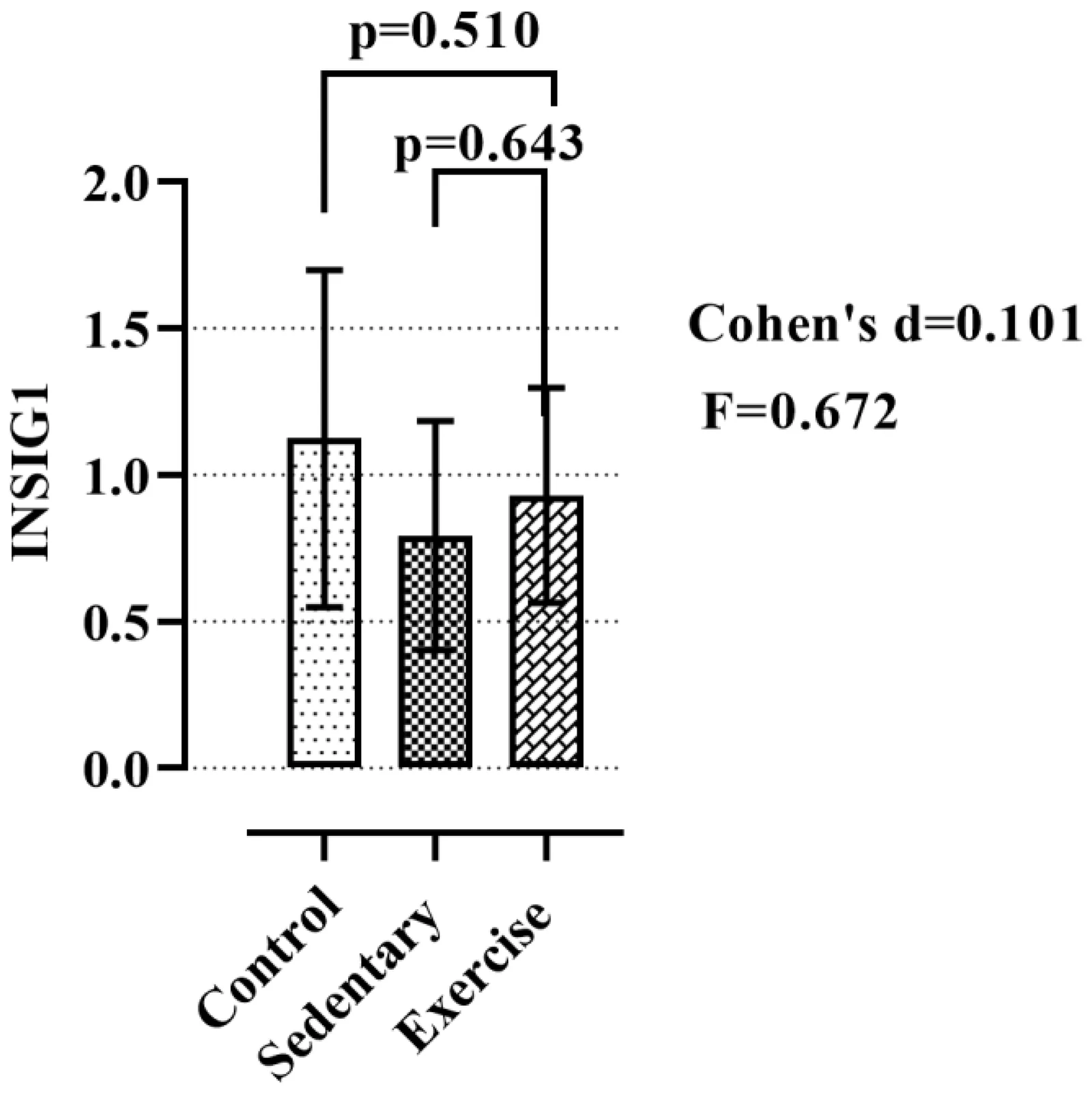
INSIG1 mRNA expression analysis across experimental groups C, Control; S, Sedentary; E, Exercise. Individual data points are displayed alongside group summary values presented as Mean ± SD (Control: 1.125 ± 0.575, Sedentary: 0.794 ± 0.391, Exercise: 0.930 ± 0.366; n = 15 rats per group). Statistical significance was evaluated using one-way ANOVA followed by Bonferroni’s *post-hoc* test for multiple comparisons. No statistically significant differences were observed between the Control and Exercise groups (p > 0.05, mean difference: 0.1946, 95% CI: -0.5708 to 0.9600) or between the Sedentary and Exercise groups (p > 0.05, mean difference: -0.1364, 95% CI: -0.9018 to 0.6290) (**Figure 3**).

## Discussion

In this study, the effects of high-intensity treadmill exercise on GLUT4, INSIG1, and TNF-α mRNA expression in soleus muscle, as well as on blood glucose levels measured at predefined time points, were investigated in diabetic rats. Blood glucose levels decreased significantly from pre-test to post-test in the exercise group; however, the post-test difference between the exercise and sedentary diabetic groups was not statistically significant. In addition, GLUT4 and TNF-α mRNA expression levels were significantly higher in the exercise group than in the sedentary diabetic group. In contrast, six weeks of exercise did not result in a statistically significant change in INSIG1 mRNA expression. Although the observed change in INSIG1 was not statistically significant, the findings may suggest that a longer exercise intervention could produce more pronounced alterations in INSIG1 expression. Given the regulatory role of INSIG1 in lipid metabolism, the duration of the exercise intervention may be an important factor influencing its transcriptional response. Therefore, longer-term exercise interventions may be warranted to further clarify the response of INSIG1 to exercise under diabetic conditions.

The higher glucose concentrations observed in the S and E groups compared with the C group across the assessment period support the successful induction and maintenance of the diabetic condition. The mixed-effects analysis revealed significant main effects of group and time, as well as a significant group × time interaction, indicating that the pattern of changes in blood glucose levels over time differed among the experimental groups. Descriptively, blood glucose levels remained relatively stable in the S group, whereas a progressive reduction was observed in the E group from week 1 to week 6. However, despite this reduction, the post-test blood glucose level in the E group did not differ significantly from that in the S group. These findings suggest that the 6-week exercise intervention may have contributed to an improvement in glycemic regulation over time, although the intervention was not sufficient to normalize blood glucose levels in diabetic rats. Previous studies have reported that both moderate- and high-intensity exercise interventions may contribute to the regulation of elevated blood glucose levels in diabetic rats (31). The frequency and intensity of exercise are known to influence glucose metabolism and glycemic responses differently. In an 18-week study examining the effects of moderate- and high-intensity exercise interventions on glucose homeostasis, moderate-intensity exercise was reported to be more effective in the measurements obtained at week 18 (32). In another study, 6 weeks of high-intensity exercise was reported to be effective in regulating blood glucose levels (33). These findings indicate that the metabolic response to exercise may vary depending on exercise intensity, duration, and frequency. Therefore, evaluating the effects of exercise solely on the basis of blood glucose levels may provide limited information, and the assessment of molecular markers involved in glucose and metabolic regulation may provide a more comprehensive understanding of the underlying adaptations. In this context, the present study also examined GLUT4, TNF-α, and INSIG1 mRNA expression in soleus muscle tissue to further characterize the molecular response to exercise under diabetic conditions.

GLUT4 is a major insulin-responsive glucose transporter that plays a central role in facilitating glucose uptake into skeletal muscle cells by translocating to the plasma membrane in response to insulin and muscle contraction. In diabetic conditions, impaired glucose uptake is associated with alterations in GLUT4 expression and translocation, which may contribute to disturbances in glucose homeostasis. Diabetes-induced rats have been reported to exhibit decreased GLUT4 expression in various tissues, including skeletal muscle (34, 35). While exercise training is known to acutely and chronically stimulate intracellular signaling pathways associated with GLUT4 translocation and muscle glucose uptake (36, 37), repeated exercise stimuli may induce sustained adaptations primarily at the transcriptional level. In the present study, the significantly higher GLUT4 mRNA expression observed in the Exercise group compared with the Sedentary group indicates that the 6-week high-intensity treadmill exercise intervention promoted an adaptive transcriptional response in skeletal muscle under diabetic conditions. These findings align with recent evidence indicating that structured exercise interventions counteract diabetes-related molecular impairments in skeletal muscle (38). However, an increase in GLUT4 mRNA expression reflects a molecular adaptation at the gene expression level and does not automatically confirm increased GLUT4 protein abundance, enhanced translocation to the plasma membrane, or improved functional glucose uptake. Therefore, the observed elevation in GLUT4 mRNA should be interpreted strictly as a muscle-specific transcriptional adaptation rather than direct evidence of restored systemic glucose transport or whole-body glycemic control.

Diabetes is known to cause elevated TNF-α expression in skeletal muscle and plasma. TNF-α has a significant effect not only on insulin signaling but also on several intracellular signaling pathways. It impairs glucose metabolism in both humans and experimental animals. It has also been reported to increase fatty acid incorporation into diacylglycerol, which may contribute to the development of TNF-α-induced insulin resistance in skeletal muscle (39). In the presence of diabetes, infiltrated inflammatory cells may release proinflammatory cytokines such as TNF-α. However, different types of exercise have been shown to reduce TNF-α levels in diabetic rats (40). Resistance exercise has also been reported to have a low-to-moderate effect on TNF-α levels (41). In line with these observations, high-intensity treadmill exercise in the present study significantly reduced TNF-α mRNA expression in the soleus muscle of diabetic rats compared with the sedentary diabetic group. This downregulation suggests that structured treadmill training may attenuate the local transcriptional inflammatory response associated with STZ-induced diabetes. While another study reported that aerobic or resistance exercise did not significantly alter TNF-α levels (42), the significant reduction observed in our study supports the potential anti-inflammatory effects of regular exercise at the skeletal muscle transcriptional level. The discrepancy among these findings in the literature may be related to differences in exercise modality, intensity, frequency, and duration, as well as differences in the experimental characteristics of the diabetic models. Furthermore, an alteration at the transcriptional level does not directly equate to changes in functional TNF-α protein abundance or systemic inflammatory status, reinforcing the need to distinguish statistical mRNA variations from definitive biological outcomes. Overall, the lower TNF-α mRNA expression observed in the exercise group indicates a potentially favorable transcriptional adaptation in skeletal muscle under diabetic conditions, although further assessment of TNF-α protein abundance and systemic inflammatory markers is warranted.

There are limited findings in the literature investigating the relationship between INSIG1 and exercise. In one study, eccentric exercise in humans was reported to alter INSIG1 mRNA expression in skeletal muscle tissue obtained by biopsy, indicating that INSIG1 may respond to exercise-related metabolic stimuli (43). In the present study, no statistically significant difference in INSIG1 mRNA expression was observed between the Exercise and Sedentary diabetic groups. This result should therefore be interpreted primarily as a null finding, indicating that the 6-week high-intensity treadmill exercise intervention did not significantly alter INSIG1 transcription in diabetic soleus muscle under the conditions examined. The absence of a statistically significant difference should not be interpreted as evidence of functional adaptation or improvement in glucose homeostasis. When considering T2DM-related metabolic traits, the absence of a significant difference in our study aligns with previous findings reporting no strong association between INSIG1 and T2DM-related parameters (18). Furthermore, Knuiman et al. (43) demonstrated that INSIG1 mRNA expression can respond to exercise acutely, with variations depending on nutritional status and biopsy timing. Given the context-dependent physiological nature of INSIG1, differences in exercise modality, duration, nutritional condition, and tissue collection timing may contribute to the variable responses observed across studies. Taken together, the present findings provide no evidence of a statistically significant alteration in INSIG1 mRNA expression following the tested exercise intervention. Although this study was conducted using an experimental rat model, our findings provide insight into molecular responses in soleus muscle under diabetic conditions. The significant increase in GLUT4 mRNA expression following exercise suggests a molecular adaptation that may support glucose utilization in skeletal muscle. In contrast, the significant reduction in TNF-α mRNA expression should be interpreted with caution, as it may reflect a tissue-specific response or the particular characteristics of the exercise protocol rather than a definitive systemic anti-inflammatory effect. Finally, INSIG1 mRNA expression showed no statistically significant change following the intervention. Overall, the results suggest that high-intensity treadmill exercise can induce gene-specific molecular responses in skeletal muscle under diabetic conditions, particularly in relation to GLUT4 and TNF-α mRNA expression. However, the biological significance of these mRNA expression changes and their relevance to glucose regulation in human diabetes require further investigation in well-controlled studies.

Crucially, rather than examining these molecular and systemic endpoints in isolation, integrating the observed pattern specifically the concurrent transcriptional upregulation of GLUT4, the attenuation of local inflammatory stress (suppressed TNF-α relative to the sedentary diabetic state), unchanged INSIG1 expression, and the lack of a statistically significant difference in post-intervention blood glucose between the exercise and sedentary diabetic groups provides a more nuanced physiological perspective. While the upregulation of GLUT4 mRNA demonstrates a robust, activity-induced adaptive drive toward enhanced glucose uptake capacity in soleus muscle, this localized molecular signal did not fully translate into systemic glycemic normalization. This apparent discordance can be explained by several factors: functional glucose clearance relies heavily on GLUT4 protein translation and translocation to the sarcolemma, processes that may remain blunted under persistent diabetic metabolic derangements despite transcriptional upregulation. Furthermore, although exercise successfully attenuated TNF-α mRNA expression compared to sedentary diabetic controls, the remaining baseline metabolic and endocrine disruptions in this STZ model likely limited the capacity of muscle-specific adaptations to override systemic hyperglycemia within a 6-week timeframe. Similarly, the unresponsiveness of INSIG1 indicates that lipid-regulatory transcriptional pathways remained largely unaltered by this specific exercise modality, suggesting that metabolic recovery in this model was restricted to specific inflammatory and contractile-responsive gene networks rather than a global transcriptional reset. Taken together, these findings highlight a clear dissociation between early localized muscle transcriptional adaptations and whole-body glycemic outcomes under diabetic conditions (44, 45).

From a translational perspective, the present findings should be interpreted within the specific characteristics and limitations of the experimental model. The STZ-induced diabetic rat model does not fully reproduce the complex pathophysiology of human T2DM, and the six-week intervention does not allow conclusions regarding long-term clinical adaptations. Thus, the present findings provide mechanistic information regarding skeletal-muscle mRNA expression responses to high-intensity treadmill exercise under diabetic conditions, but they cannot be directly extrapolated to clinical improvements in patients with T2DM. Further studies assessing protein-level responses, insulin sensitivity, whole-body glucose metabolism, systemic inflammatory markers, and longer-term exercise adaptations are needed to determine whether these molecular responses translate into functional and clinically meaningful benefits in humans.

## Limitations of the study

The present study has several limitations that should be considered when interpreting the findings. First, the high-intensity treadmill exercise intervention was limited to 6 weeks; therefore, the effects of longer-term exercise on molecular adaptations could not be determined. Second, physiological or stress-related responses associated with the experimental procedures and exercise intervention including direct physiological indices of exercise intensity such as blood lactate concentration or maximum oxygen consumption were not assessed. Additionally, food intake, water consumption, and continuous body-weight dynamics were not systematically tracked throughout the intervention period, which limits a comprehensive assessment of overall energy balance in this diabetic model. In addition, functional metabolic assessments such as insulin tolerance tests (ITT), glucose tolerance tests (GTT), and comprehensive lipid endpoints were not performed, which limited our ability to evaluate changes in insulin sensitivity, lipid metabolism, and glycemic control in greater detail.

Furthermore, only GLUT4, TNF-α, and INSIG1 mRNA expression was examined in soleus muscle tissue; therefore, the findings cannot be generalized to other tissues or to protein-level responses. In particular, functional protein-level validations including total GLUT4 protein abundance, GLUT4 translocation to the plasma membrane, TNF-α protein levels, and upstream/downstream key targets of the insulin signaling cascade were not determined, thereby constraining the mechanistic depth of our molecular interpretations. Moreover, circulating inflammatory markers were not assessed; thus, the observed suppression of TNF-α mRNA cannot be directly extrapolated to systemic inflammatory status. Another design constraint is that this study was conducted exclusively on male rats to eliminate estrous cycle variability, which limits the generalizability of these findings to female sex physiology. Regarding the longitudinal analysis of blood glucose trajectory, the sample size represents a statistical constraint when capturing subtle temporal shifts.

Another methodological limitation is that GAPDH was used as a single reference gene for normalization of target gene expression without independent validation of its expression stability across the experimental groups. Although GAPDH is widely used as a reference gene in skeletal muscle and metabolic research, the stability of housekeeping genes may vary depending on the tissue, disease state, and experimental intervention. Therefore, the possibility that diabetes or exercise-related metabolic alterations may have influenced GAPDH expression cannot be completely excluded. Future studies should evaluate multiple candidate reference genes and validate their stability under the specific experimental conditions before normalization.

An additional important limitation concerns the experimental diabetes model. Diabetes was induced by a single intraperitoneal injection of streptozotocin (STZ; 60 mg/kg), without the use of high-fat feeding or nicotinamide pretreatment. Because STZ primarily induces pancreatic beta-cell injury, this protocol may produce a diabetic phenotype characterized predominantly by impaired insulin secretion rather than the combination of insulin resistance and beta-cell dysfunction typically observed in T2DM. In the present study, insulin concentrations and direct measures of insulin sensitivity, such as HOMA-IR, glucose tolerance, or insulin tolerance, were not assessed. Therefore, the presence or extent of insulin resistance could not be established, and the present experimental model should not be interpreted as a complete representation of human T2DM pathophysiology. Accordingly, the findings should be interpreted specifically in the context of STZ-induced diabetes.

## Conclusion

In conclusion, a 6-week high-intensity treadmill exercise regimen induced distinct, tissue-specific transcriptional adaptations in the soleus muscle of STZ-induced diabetic rats, characterized by a significant upregulation of GLUT4 mRNA expression and a significant attenuation of TNF-α mRNA expression compared with the sedentary diabetic group. In contrast, INSIG1 mRNA expression showed no statistically significant change, indicating that INSIG1 transcription was unresponsive to the tested intervention. Importantly, although blood glucose levels significantly decreased from pre-test to post-test within the exercise group, the post-test blood glucose level did not differ significantly from that of the sedentary diabetic group. Taken together, these findings demonstrate that high-intensity exercise effectively modulates specific gene networks involved in muscle glucose transport and local inflammatory regulation under diabetic conditions; however, these localized transcriptional shifts should be interpreted strictly as gene expression adaptations rather than evidence of restored systemic glucose homeostasis or clinical metabolic recovery.

## Data Availability

The raw data supporting the conclusions of this article will be made available by the authors, without undue reservation.
